# Sexual Orientation, Peer Influence, Body Dissatisfaction, and Eudaimonic Well-Being in Italian Men

**DOI:** 10.3389/fpsyg.2019.01843

**Published:** 2019-08-08

**Authors:** Camilla Matera, Amanda Nerini, Cristina Stefanile

**Affiliations:** ^1^Department of Education, Languages, Intercultures, Literatures and Psychology, University of Florence, Florence, Italy; ^2^Department of Health Sciences, University of Florence, Florence, Italy

**Keywords:** sexual orientation, peer influence, body dissatisfaction, eudaimonic well-being, men

## Abstract

Having a lean and athletic physique is increasingly important for Italian men. The purpose of this cross-sectional study was to analyze the relationship between men’s dissatisfaction with muscularity and well-being, conceptualized in terms of the realization of one’s true potential and the experience of purpose or meaning in life (i.e., eudaimonic well-being), considering also the role of sexual orientation and peer influence. Participants (385 Italian men with a mean age of 28.61 years, *SD* = 9.65) completed a questionnaire assessing the variables of interest. Path analysis indicated that sexual orientation was linked to eudaimonic well-being via muscularity dissatisfaction. Teasing about muscularity predicted men’s eudaimonic well-being both directly and via muscularity dissatisfaction. Peer attributions and appearance conversations predicted well-being through the mediation of athletic internalization and body dissatisfaction. Peer attributions, but not appearance conversations, were also directly linked to well-being. To improve broad aspects of men’s well-being, prevention and treatment programs should be directed to increase men’s ability to resist different forms of pressure, including that of their peers. These kinds of programs could be especially useful for gay men, who are more likely to experience body dissatisfaction and, in turn, poor psychological functioning.

## Introduction

Having an athletic physique is increasingly important for men, who aspire to a body ideal that is more muscular than their actual one (Frederick et al., 2007; Tiggemann et al., 2007). The internalization of appearance ideals (Grammas and Schwartz, 2009; Daniel and Bridges, 2010; Cramblitt and Pritchard, 2013) has been identified as a significant predictor of men’s body dissatisfaction (Karazsia and Crowther, 2010); the more men internalize appearance societal ideals as personal standards, the higher body dissatisfaction they are likely to experience (Tylka, 2011). Even in the Italian context, the body standard for men is more muscular than the average male body shape (Zelli et al., 2010; Gilli and Ruspini, 2014). The traditional Italian stereotype of men is one of strength and virility; in this context, muscularity has been traditionally linked to the concept of masculinity (Fabris et al., 2017). Nevertheless, nowadays alongside the traditional image of the virile and powerful man, a new type of masculinity has emerged (Boni, 2004, 2007), the so-called ‘metrosexual’ man, who is particularly concerned with the care of the body, is a strong consumer of cosmetics, and is interested in aesthetics (Capellani et al., 2014). Italian fashion has contributed to the proposition of a more flexible and less rigidly regulated model of masculinity (Mora, 2007); in general, great attention to the body is paid by Italian men (Ruspini, 2007).

Some research suggests that men’s body image is linked to their well-being (Swami et al., 2018). Men who are excessively concerned with their body are more likely to experience low self-esteem, negative affect, distress, depression, and use of performance-enhancing substance, with deleterious effects on their general psychological functioning (Olivardia et al., 2004; Grossbard et al., 2009).

Most studies that have examined the relationship between body dissatisfaction and well-being have focused on indices of hedonic well-being, such as pleasure and happiness (Ryan and Deci, 2001). Alternatively, eudaimonic well-being, conceptualized in terms of the realization of one’s true potential (Ryff and Keyes, 1995) and the experience of purpose or meaning in life (Ryff, 1989), has been rarely considered with respect to body image. An exception was a recent study by Swami et al. (2018), who found that positive body image was positively associated not only with the presence of positive emotions and overall satisfaction with life, but also with eudaimonic well-being, assessed in terms of autonomy, sense of personal growth, and social functioning. Specifically, body appreciation emerged as a significant predictor of emotional (hedonic), psychological, and social (eudaimonic) well-being. To identify significant predictors of eudaimonic well-being is especially important if we consider that it provides protection against disease, disability, morbidity, and early mortality (Ryff, 2013, 2017), consisting of facets that relate to the individuals’ both psychological (i.e., flourishing and self-realization) and social life (i.e., functioning well in the social realm).

The present study provided a novel contribution to the literature by further analyzing the relationship between men’s dissatisfaction with muscularity and eudaimonic well-being, considering also the role of sexual orientation and peer influence. We aimed to test a model in which sexual orientation and different forms of peer influence would predict men’s eudaimonic well-being via the mediation of both the internalization of athletic ideals and dissatisfaction with muscularity.

Even though greater attention to one’s body is paid by men in general, the gay male culture especially emphasizes physical appearance and places great importance on physical attractiveness (Morgan and Arcelus, 2009; Dakanalis et al., 2012). Several studies showed that gay men are more concerned with muscularity than heterosexual men (Peplau et al., 2009; Yean et al., 2013; Frederick and Essayli, 2016), also in the Italian context (Cella et al., 2013; Nerini et al., 2016). According to the minority stress model (Meyer, 1995, 2003) gay men might perceive greater pressure to conform to some widespread norms and ideals in order to feel more accepted by the overall society. Indeed, Kimmel and Mahalik (2005) found that conformity to masculine norms was associated with gay men’s distress if their body did not meet the physically powerful masculine ideal. Peplau et al. (2009) were among the few researchers who examined body image and quality of life in both heterosexual and gay men: gay men reported less positive evaluation of their appearance, higher weight concern, and less positive effects of their body image on their quality of life. We argue that similar findings could emerge with respect to men’s eudaimonic well-being; eudaimonic happiness is achieved when individuals face challenges in order to realize their unique talents (Ryff and Keyes, 1995) and are functioning well in the social realm (Keyes, 1998). Due to the great emphasis posed by the gay male culture on physical appearance (Morgan and Arcelus, 2009; Dakanalis et al., 2012), gay men might be more likely to internalize widespread appearance ideals and to perceive some discrepancy between their actual and ideal body (Kimmel and Mahalik, 2005). Such a focus on appearance might enhance the importance given to superficial aspects of existence, which might be a limit for the realization of individuals’ true potential and for optimal functioning in society.

Different factors might enhance the importance that men give to appearance, among which peers play a relevant role (Karazsia and Crowther, 2009; Tylka, 2011; Matera et al., 2018). Teasing seems to be directly linked to body dissatisfaction (van den Berg et al., 2007; Puhl et al., 2017), emotional health problems (Eisenberg et al., 2003), and low self-esteem (Nowell and Ricciardelli, 2008) among men. Peer attributions about the importance of weight and shape for popularity (Lieberman et al., 2001) and appearance conversations with friends (Jones et al., 2004) are less studied forms of peer influence. Peer attributions can be defined as the extent to which individuals believe that their friends attribute importance to appearance, so that appearance might be perceived as a useful means to gain popularity (Lieberman et al., 2001). Appearance conversations with friends refer to everyday conversations about looks, appearance enhancements, and attractiveness (Jones, 2004). Some studies carried out on Italian women showed that these forms of influence were differently linked to body image. Whereas teasing was directly associated with body dissatisfaction, the effect of both appearance conversations and peer attributions was mediated by the internalization of appearance societal ideals (Matera et al., 2013; Stefanile et al., 2015).

Gay men feel greater pressure from their peers to achieve an ideal body than heterosexual men (McArdle and Hill, 2009). Tylka and Andorka (2012) found that internalization of the mesomorphic ideal, appearance comparison, muscularity dissatisfaction, and body fat dissatisfaction connected different sources of social influence, such as family and friends, to gay men’s body image and/or body change behaviors. To the best of our knowledge, only one study examined the relationship between appearance conversations with friends and men’s body image considering also their sexual orientation (Jankowski et al., 2014). Nevertheless, no indicators of well-being were included in this study. The link between peer attributions and well-being among men appears almost unexplored. It is our contention that men who feel higher levels of peer influence, in terms of teasing, appearance conversations, and peer attributions, might be more likely to focus highly on appearance, which could hamper the development of their true self and the achievement of a sense of meaning and purpose in life. Either the internalization of appearance ideals or feelings of dissatisfaction with one’s body could be partially responsible for such a relationship between peer influence and eudaimonic well-being.

This study tested a model in which sexual orientation was posited as a predictor of men’s eudaimonic well-being through athletic internalization and muscularity dissatisfaction (Hypothesis 1). Together with sexual orientation, different forms of peer influence were posited as predictors of men’s eudaimonic well-being: teasing about one’s muscularity was hypothesized to predict men’s eudaimonic well-being, both directly and indirectly, through the mediation of muscularity dissatisfaction (Hypothesis 2), while appearance conversations with friends and peer attributions were hypothesized to predict eudaimonic well-being both directly and indirectly, via athletic internalization and muscularity dissatisfaction (Hypothesis 3). Men’s body mass index (BMI) on men’s internalization of athletic ideals and muscularity dissatisfaction was included in the model in order to control for its effects.

## Materials and Methods

### Participants

Participants were 385 Italian men with a mean age of 28.61 that ranged from 18 to 76 years (*SD* = 9.65) and a mean BMI of 23.33 (*SD* = 3.04). Based on BMI, participants could be classified as follows: 1% underweight, 78.7% normal weight, 16.1% overweight, and 4.2% obese. Most participants (97.9%) were not married. Only 1.6% were married, and 0.5% were separated or divorced. Participants had studied for 15.66 years on average (*SD* = 3.43). Specifically, 53.8% had completed high school (13th grade), 17.4% had a graduate degree, 16.1% had an undergraduate degree, 10.6% had finished middle school (8th grade), and 2.1% had some other type of qualification.

### Measures

#### Body Mass Index

Participants’ self-reported height and weight were collected to calculate BMI.

#### Demographics

Participants provided their sociodemographic details: age, educational level (measured in terms of years of education/schooling), and marital status (not married, married, separated/divorced, other).

#### Sexual Orientation

A five- point Kinsey-like scale (Kinsey et al., 1948) was used to assess individuals’ sexual orientation in terms of sexual attraction. This is a single-item scale with scores ranging from 1 to 5, with 1 indicating that someone was *Exclusively heterosexual, never homosexual*; 5 indicating that someone was *Exclusively homosexual, never heterosexual*; and intermediate scores indicating degrees in between (2 = *Predominantly heterosexual, incidentally homosexual*; 3 = *Equally heterosexual and homosexual*; 4 = *Predominantly homosexual, incidentally heterosexual*). With respect to sexual orientation, 62.9% described themselves as exclusively heterosexual, 27% as exclusively homosexual, and 10.1% as predominantly homosexual, incidentally heterosexual. No respondents identified themselves as predominantly heterosexual, incidentally homosexual, or equally heterosexual and homosexual. For the purposes of this study, participants who described themselves as exclusively or predominantly homosexual were regrouped in the same category, so that sexual orientation was a dichotomous variable (1 = heterosexual men, *n* = 242; 2 = gay men, *n* = 143). This procedure is in line with much contemporary sex research, in which sexual orientation has been initially assessed as a continuum, ranging from exclusively opposite-sex to exclusively same-sex attraction, with degrees of non-exclusivity in between—usually rated on a 5- or 7-point Kinsey-like scale— and subsequently the continuous construct has been collapsed into discrete, mutually exclusive categories (Savin-Williams, 2016).

#### Peer Influence

To assess peer influence, we adopted three measures which were found to be valid and reliable with Italian men (Stefanile et al., 2010). When applied in the Italian context these scales presented good internal consistency, with alpha values ranging from 0.83 to 0.90 (Matera et al., 2018).

The Appearance Conversations with Friends Scale (Jones et al., 2004) assessed how often participants were involved in conversations with friends about their appearance, using 3 items (e.g., ‘My friends and I talk about the size and shape of our bodies’) rated on a 5-point, Likert-type scale (1 = *Never*, 5 = *Always*). In the present study, internal consistency was good (*a* = 0.88).

The Peer Appearance Criticism–Muscularity assessment (Jones et al., 2004) was used to assess teasing related to muscularity (e.g., ‘Boys say I would look better if I were more muscular’). This is a 3-item assessment ranging from 1 (*never*) to 5 (*always*). Internal consistency in the present study was good (*a* = 0.84).

The Peer Attribution Scale, developed by Lieberman (2000), was used to measure attributions related to appearance made by male friends. This is a 4-item scale (e.g., ‘If I was better looking I would be more popular’), ranging from 1 (false) to 6 (true). This scale has been found to be unidimensional, and evidence of reliability has been accrued within the Italian context (Stefanile et al., 2010). Internal consistency was acceptable in the present study (*a* = 0.75).

#### Internalization of Athletic Ideals

To assess the internalization of athletic ideals promoted by the media we used the Internalization-Athletic subscale of the Italian version for males (Nerini et al., 2011) of the Sociocultural Attitudes toward Appearance Scale-3 (Thompson et al., 2004). The scale was composed of five items (e.g., ‘I try to look like athletes’) rated on a 5-point, Likert-type scale (1 = *Definitively disagree*, 5 = *Definitively agree*). Exploratory factor analyses of the scale supported its structural validity (Nerini et al., 2011) and the reported internal consistency was 0.84. In the present study, the alpha value was very good (*a* = 0.88).

#### Muscularity Dissatisfaction

To assess men’s body dissatisfaction, the Italian version (Stefanile et al., 2016) of the Dissatisfaction with Muscularity subscale of the Male Body Attitudes Scale (Tylka et al., 2005) was used. This subscale is composed of 10 items (e.g., ‘I think my arms should be more muscular’) rated on a 6-point scale (1 = *Never*, 6 = *Always*). Exploratory factor analyses on the Italian version of the scale supported the structural validity of the Dissatisfaction with Muscularity subscale (Stefanile et al., 2016), for which the reported internal consistency was 0.88. In this study, the alpha of this subscale was very good (*a* = 0.87).

#### Well-Being

The Italian version (Sirigatti et al., 2009) of the Ryff’s Psychological Well-Being Scales (Ryff, 1989) was used to assess participants’ eudaimonic well-being in terms of autonomy, personal growth, self-acceptance, purpose in life, positive relations and environmental mastery. This version is composed of 18 items (e.g., ‘I judge myself by what I think is important, not by the values of what others think is important’) rated on a 6-point scale (1 = *Definitively disagree*, 6 = *Definitively agree*). Exploratory and confirmatory factor analyses supported the structural validity of the scale (Sirigatti et al., 2009). When applied in the Italian context the scale presented good internal consistency (*a* = 0.86; Baroni et al., 2018). For the present study, its reliability was acceptable (*a* = 0.72).

### Procedure

This study was carried out in multiple cities of central Italy. A non-probabilistic procedure was followed to recruit the participants, who were invited to complete a questionnaire on attitudes toward one’s body image. No incentives were offered to them. Some participants were approached by a researcher from public places, such as universities, libraries, leisure centers, reading rooms. With regard to university settings, at the end of some lessons a researcher asked students if they wanted to take part in the study; they were told that no incentives were offered for this participation and were left completely free to decide if they wanted to participate. The ones who took part in the study remained in their classroom, while the others left it. Students who agreed to participate were properly distanced, so that their privacy was maintained during the administration. Some other participants were recruited through a range of well-known associations serving sexuality groups within the Italian context. We first contacted some of these associations in order to present our study; successively some appointments were scheduled for the administration of the questionnaire.

For each administration we specified that the inclusion criteria were to be male and at least 18 years old. Participants’ sexual orientation was assessed only *a posteriori* (self-reported measure). Data were collected via pen and paper. Prior to completing the questionnaire, all of the participants provided informed consent. Questionnaires were anonymous, and no personal identifying data were recorded. Men who consented to participate were asked to complete the questionnaire individually or in small groups. When the questionnaire was administered in small groups (e.g., during university courses), full respect for privacy was guaranteed thanks to the use of spaces that allowed to adequately distance the respondents. A researcher took care that privacy was maintained throughout the administration. The survey took about 20 min to complete. All participants were thanked and debriefed after they had returned their questionnaires to the researcher. The study procedures were approved by the Ethical Committee of the University of Florence.

### Data Analyses

First of all, *t-*test for independent samples was performed to examine differences between gay and heterosexual men with regard to both demographic (age, education, and BMI), and psychosocial variables (appearance conversations with friends, peer attributions, teasing on muscularity, athletic internalization, dissatisfaction with muscularity, and eudaimonic well-being). Descriptive and correlational analyses (Pearson’s *r*) were performed, followed by path analysis (AMOS software version 20). The maximum likelihood method of estimation was applied. Bootstrapping was used to test mediation by estimating the presence and size of the indirect (i.e., mediated) effects (Preacher and Hayes, 2008). The data set did not contain missing values, and were normally distributed (skewness < 1.30, kurtosis < 1.58). Model evaluation was performed according to the criteria suggested by Schermelleh-Engel et al. (2003). Comparative fit index (CFI), incremental fit index (IFI) and normed fit index (NFI) values between 0.95 and 0.97 suggest an acceptable model fit, while values greater than 0.97 indicate a good model fit. For the root mean square error of approximation (RMSEA), values between 0.05 and 0.08 suggest an acceptable fit, while values lower than 0.05 suggest a good fit. With respect to the standardized root mean square residual (SRMR), well-fitting models obtain values lower than 0.05. Fit is considered good if the χ^2^*/df* is at or below 2, while it is considered acceptable if it is between 2 and 3.

## Results

Table 1 shows the descriptive statistics for the research variables. On average, the participants presented moderate levels of body dissatisfaction and good eudaimonic well-being.

**TABLE 1:** Descriptive statistics.

	***Min***	***Max***	***M***	***SD***
Appearance conversations with friends	1.00	5.00	2.27	1.02
Peer attributions	1.00	6.00	1.90	1.04
Teasing on muscularity	1.00	5.00	1.95	1.00
Athletic internalization	1.00	5.00	2.52	1.08
Dissatisfaction with muscularity	1.00	6.00	3.23	1.08
Eudaimonic well-being	1.83	5.50	4.27	0.57

The *t*-test showed significant differences between gay and heterosexual men with respect to age and education: gay men reported to be older and to have a higher educational level than heterosexual participants. Neither age nor education were significantly correlated with participants’ dissatisfaction with muscularity and psychological well-being. However, participants’ education was significantly correlated with athletic internalization; age was significantly correlated with BMI, appearance conversations with friends, and teasing on muscularity.

Significant differences between the two groups were found with regard to peer attributions, athletic internalization, and muscularity dissatisfaction. For these variables, gay men reported higher scores than heterosexual men (Table 2).

**TABLE 2:** Comparisons between gay and heterosexual men.

	***M (SD) gay men (n* = *143)***	***M (SD) heterosexual men (n* = *242)***	***t*_(383)_**	***Cohen’s d***
Age	36.97 (10.31)	23.67 (4.46)	17.50^∗∗∗^	1.67
Education	17.01 (4.12)	14.86 (2.65)	6.22^∗∗∗^	0.062
BMI	23.38 (3.54)	23.29 (2.72)	0.26	0.003
Appearance conversations with friends	2.35 (1.07)	2.21 (0.98)	1.27	0.014
Peer attributions	2.10 (1.11)	1.78 (0.98)	2.93^∗∗^	0.030
Teasing on muscularity	1.95 (0.98)	1.95 (1.01)	–0.08	0.000
Athletic internalization	2.70 (1.15)	2.41 (1.03)	2.61^∗∗^	0.026
Dissatisfaction with muscularity	3.47 (1.20)	3.10 (0.98)	3.26^∗∗^	0.034
Eudaimonic well-being	4.24 (0.56)	4.29 (0.58)	–0.90	0.009

^*^p < 0.05, ^∗∗^*p* < 0.01, and ^∗∗∗^*p* < 0.001.

Almost all of the research variables were significantly intercorrelated (see Table 3).

**TABLE 3:** Correlations among variables.

	**1.**	**2.**	**3.**	**4.**	**5.**	**6.**	**7.**	**8.**	**9.**
(1) Sexual orientation	–								
(2) Appearance conversations with friends	0.070	–							
(3) Peer attributions	0.138^∗∗^	0.367^∗∗∗^	–						
(4) Teasing on muscularity	0.002	0.502^∗∗∗^	0.350^∗∗∗^	–					
(5) Athletic internalization	0.138^∗∗^	0.409^∗∗∗^	0.280^∗∗∗^	0.302^∗∗∗^	–				
(6) Dissatisfaction with muscularity	0.166^∗∗^	0.367^∗∗∗^	0.292^∗∗∗^	0.524^∗∗∗^	0.410^∗∗∗^	–			
(7) Eudaimonic well-being	–0.036	–0.188^∗∗∗^	–0.346^∗∗∗^	–0.305^∗∗∗^	–0.204^∗∗∗^	–0.308^∗∗∗^			
(8) Age	0.667^∗∗∗^	−0.109^*^	0.042	–0.170^∗∗^	–0.076	–0.014	0.006		
(9) Education	0.303^∗∗∗^	0.039	0.078	0.003	0.136^∗∗^	0.076	–0.007	0.312^∗∗∗^	
(10) BMI	0.013	–0.046	0.112^*^	−0.124^*^	–0.154^∗∗^	–0.234^∗∗∗^	0.051	0.265^∗∗∗^	0.06

^*^p < 0.05, ^∗∗^*p* < 0.01, and ^∗∗∗^*p* < 0.001.

Path analysis was applied to test the hypothesized model. Due to the differences we observed between heterosexual and gay participants, also age and education were included in the model in order to control for their effects. Based on the results of the correlational analyses, education was posited as a covariant with sexual orientation and as a predictor of athletic internalization; age was posited as a covariant with sexual orientation, BMI, appearance conversations with friends, and teasing on muscularity.

**FIGURE 1:**
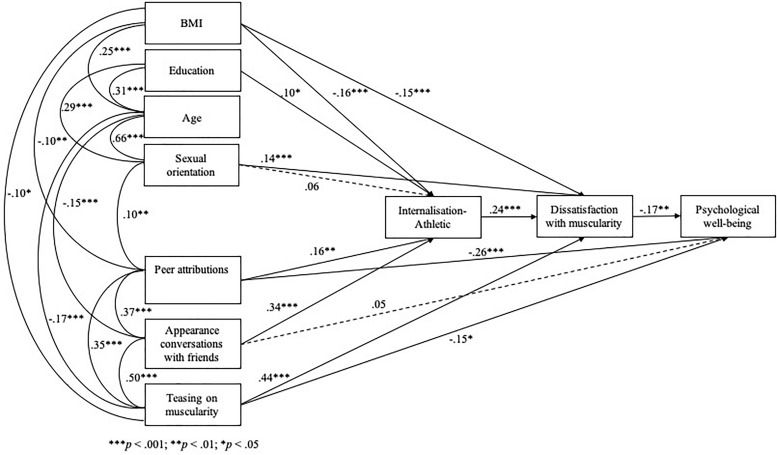
Final model of variables predicting psychological well-being in men (hatched arrows represent non-significant paths.

In line with the results of the correlational analyses, path analysis findings showed that, age was a significant covariant with sexual orientation, BMI, appearance conversations with friends, and teasing on muscularity; education was a significant covariant with sexual orientation and age; sexual orientation was a significant covariant with age, education, and peer attributions; BMI was a significant covariant with age, peer attributions and teasing on muscularity. The model had a very good fit to the data (χ^2^ = 22.42, *p* = 0.20; χ^2^*/df* = 1.12; CFI = 1.00; NFI = 0.98; IFI = 1.00; SRMR = 0.03; RMSEA = 0.02; RMSEA 90% CI = 0.00, 0.05) and explained a satisfactory amount of variance of men’s eudaimonic well-being (17%). The only paths that did not emerge as significant were the one between sexual orientation and athletic internalization and the one between appearance conversations with friends and well-being (see Figure 1).

In line with Hypothesis 1, the results of the bootstrapping procedure showed that the indirect effect of sexual orientation on eudaimonic well-being via muscularity dissatisfaction was significant (95% CI = −0.019, −0.002). In line with Hypothesis 2, teasing about muscularity predicted men’s well-being both directly and indirectly, via body dissatisfaction (95% CI = −0.073, −0.017); higher teasing related to men’s body shape was associated with higher dissatisfaction with muscularity, which in turn was related to lower eudaimonic well-being. With regard to Hypothesis 3, the indirect effects of both peer attributions (95% CI = −0.010, −0.001) and appearance conversations (95% CI = −0.019, −0.003) on eudaimonic well-being (via internalization and muscularity dissatisfaction) were significant. The more men talked to friends about appearance and believed their peers attributed importance to attractiveness, the more they were likely to internalize athletic ideals, to feel dissatisfied with their muscularity and, in turn, to experience poorer eudaimonic well-being. Peer attributions, but not appearance conversations, were also directly linked to participants’ well-being.

## Discussion

This study provided a novel contribution to the literature on men’s body image by showing a significant relationship between men’s muscularity dissatisfaction and eudaimonic well-being, considering the role of sexual orientation and different forms of peer influence. Feelings of dissatisfaction with one’s muscularity were negatively associated with men’s perceptions of autonomy, purpose in life, mastery, growth, self-acceptance, and positive relations with others (Ryff, 1989). Participants presented good eudaimonic well-being but moderate levels of dissatisfaction with muscularity.

The research hypotheses were partially confirmed. Sexual orientation was associated with men’s eudaimonic well-being through body dissatisfaction. Contrary to previous international findings (Tylka and Andorka, 2012), the mediational role of the internalization of athletic ideals was not confirmed. Gay men were more likely to be dissatisfied with their muscularity, probably due to the high emphasis on body appearance in the Italian gay community (Cella et al., 2013; Nerini et al., 2016). In fact, gay men considered that appearance is important for being popular among friends, more than heterosexual men did. As a consequence, Italian gay men might feel pressured to assume roles and characteristics that are associated with their biological sex (Baiocco et al., 2014). Italian gay men might feel especially pressured to conform to cultural expectations of physical attractiveness, compared to heterosexuals, because they might be afraid of becoming targets of discrimination if they violate traditional masculine norms (Dakanalis et al., 2012; Lingiardi et al., 2012). Similar findings emerged also in other sociocultural contexts, such as the United States: gay men are concerned about having a muscular body (Peplau et al., 2009; Yean et al., 2013; Frederick and Essayli, 2016), probably because they believe they are more likely to be socially accepted if they adhere to the stylized heavily mediated form of masculinity (Duncan, 2010). It might be that core components of eudaimonic well-being, such as autonomy or self-acceptance, are threatened when an individual believes he is not free to be the way he is, and that he instead has to conform to widespread and difficult-to-achieve aesthetic ideals. In line with the minority stress model, social stress might have a strong impact in the lives of people belonging to minority categories, such as gay men (Meyer, 1995, 2003). This kind of stress can be significantly associated with both body image dissatisfaction and masculine body ideal distress among gay men, for whom being masculine might enhance feelings of acceptance within the dominant heterosexual society (Kimmel and Mahalik, 2005). Previous studies conducted in Italy with gay and heterosexual men showed that gay men tended to objectify their body more than heterosexuals, presented higher levels of disordered eating behaviors, and were found to be more depressed (Dakanalis et al., 2012).

As hypothesized, eudaimonic well-being was significantly predicted by different forms of peer influence. Beside teasing (van den Berg et al., 2007; Schuster et al., 2013; Vartanian et al., 2013), even less-studied forms of peer influence, such as appearance conversations with friends and peer attributions, were found to be related to well-being in a different and peculiar manner. These findings confirmed the importance of considering peer influence as being multifaceted (Matera et al., 2018). As hypothesized, men who felt such pressures from their peers were more likely to feel dissatisfied with their body; this focus on aesthetics might limit men’s efforts to realize their individual talents and true potential. As predicted, teasing had a direct link to men’s muscularity dissatisfaction, whereas the effect of appearance conversations and peer attributions was partially mediated by the internalization of athletic ideals. By talking about the body and by attributing much importance to appearance for popularity, friends seem to encourage the internalization of some specific standards of appearance, which probably results in increased body dissatisfaction, and, in turn, in lower readiness to respond to life challenges and daily stresses.

We believe the findings from this study have potential implications for both prevention and treatment efforts aimed at fostering men’s well-being. To improve broad aspects of men’s well-being, prevention and treatment programs should be directed to increase men’s ability to resist different forms of pressure, including that of their peers. Men should be encouraged to develop some form of disapproval regarding their friends’ beliefs when excessive importance is given to appearance. Additionally, they might learn to be comfortable with their body image even if they are faced with criticism and teasing from their peers These kinds of programs could be especially useful for gay men, who are more likely to believe that their peers consider appearance important for social acceptance and to experience dissatisfaction with their muscularity.

Programs that target body-ideal internalization in men through different techniques, such as cognitive dissonance (Brown and Keel, 2015; Brown et al., 2017; Jankowski et al., 2017), might be useful not only to reduce body dissatisfaction and eating pathology, but also to enhance eudaimonic well-being. These programs might take place in individual or group sessions or might be a part of outreach programing to educate men about the potential connection between peer influence, internalized appearance ideals, body image concerns, and well-being.

These findings are especially important if we consider that eudaimonic well-being consists of facets relating to the individuals’ psychological (i.e., flourishing and self-realization) and social life (i.e., functioning well in the social realm). Eudaimonic well-being also matters for health, providing protection against disease, disability, and early mortality (Ryff, 2013). Indeed, much research has consistently documented health benefits (reduced morbidity, extended longevity) among older adults who remain purposefully engaged (Ryff, 2017).

There are some limitations to the present study that should be considered. First, our sample was not representative of Italian men; participants were recruited through a non-probabilistic procedure, which could undermine the generalizability of our findings to that of the entire nation. Second, due to the cross-sectional nature of this research, causal inferences cannot be made. Third, self-report questionnaires were used. Future studies could add independent reports obtained from peers (such as focus groups). Fourth, in our study gay and heterosexual men differed in age and educational level; even though we controlled for these variables in our statistical analyses, future research could recruit more homogeneous groups with regard to these demographic factors. Fifth, we included in the sample individuals who reported to be male, but we did not ask for transgender identity. Future research could add some questions about gender identification in order to examine potential differences between transgender and cisgender men. Sixth, we did not account for previous mental health diagnoses and social problems. Moreover, we treated sexual orientation as a dichotomous variable; future studies could consider it as a continuum, using also more detailed and multi-dimensional measures. Finally, the present study was not exhaustive in studying the potential factors that might influence eudaimonic well-being; indeed, the variance explained by the model was not high; this could be due to the complexity of the construct of eudaimonic well-being, which can be influenced by a great deal of individual, social and contextual factors. Future study could examine further variables that might be associated with men’s body image and eudaimonic well-being, such as the influence of one’s family and partner and the tendency to compare one’s body to the one of others. It could also be interesting to test our model among gay and heterosexual men, respectively, in order to highlight potential differences between the two groups.

## Data Availability

The datasets generated for this study are available on request to the corresponding author.

## Ethics Statement

The studies involving human participants were reviewed and approved by Ethical Committee of the University of Florence. The patients/participants provided their written informed consent to participate in this study.

## Author Contributions

All authors listed have made a substantial, direct and intellectual contribution to the work, and approved it for publication.

## Conflict of Interest Statement

The authors declare that the research was conducted in the absence of any commercial or financial relationships that could be construed as a potential conflict of interest.
